# Association of Long Non-Coding RNA HOTAIR Polymorphisms with Cervical Cancer Risk in a Chinese Population

**DOI:** 10.1371/journal.pone.0160039

**Published:** 2016-07-28

**Authors:** Liangsheng Guo, Xueguan Lu, Lijun Zheng, Xianying Liu, Min Hu

**Affiliations:** 1 Department of Obstetrics and Gynecology, The Second Affiliated Hospital of Soochow University, Suzhou, China; 2 Department of Radiation Oncology, Fudan University Shanghai Cancer Center, Shanghai Medical College, Fudan University, Shanghai, China; 3 Department of Radiotherapy & Oncology, The Second Affiliated Hospital of Soochow University, Suzhou, China; 4 Department of Ultrasound, The Second Affiliated Hospital of Soochow University, Suzhou, China; 5 Clincal Skills Training Center, The Second Hospital of Jilin University, Changchun, China; Shandong Cancer Hospital and Institute Affiliated to Shandong University, CHINA

## Abstract

Long non-coding RNAs (lncRNAs), HOTAIR has been reported to be upregulated in cervical cancer development and progression. However, SNPs (single nucleotide polymorphisms) in the lncRNAs and their associations with cervical cancer susceptibility have not been reported. In the current study, we hypothesized that SNPs within the lncRNA HOTAIR may influence the risk of cervical cancer. We performed a case-control study including 510 cervical cancer patients (cases) and 713 cancer-free individuals (controls) to investigate the association between three haplotype-tagging SNPs (rs920778, rs1899663 and rs4759314) in the lncRNA HOTAIR and the risk of cervical cancer. We found a strong association between the SNP rs920778 in the intronic enhancer of the HOTAIR and cervical cancer (*P*<10^−4^). Moreover, the cervical cancer patients with homozygous TT genotype were significantly associated with tumor-node-metastasis (TNM) stage. In *vitro* assays with allele-specific reporter constructs indicated that the reporter constructs bearing rs920778T allele conferred elevated reporter gene transcriptional activity when compared to the reporter constructs containing rs920778C allele. Furthermore, HOTAIR expression was higher in cervical cancer tissues than that in corresponding normal tissues, and the high expression was associated with the risk-associated allele T. In summary, our studies provide strong functional evidence that functional SNP rs920778 regulates HOTAIR expression, and may ultimately influence the predisposition for cervical cancer.

## Introduction

Worldwide, cervical cancer is the second most-common cancer and fourth most frequent cause of death from cancer among females. It was estimated that there were approximately 528,000 new cases and 266,000 deaths in 2012. Epidemiology studies have demonstrated that high-risk human papillomaviruses (HPVs), smoking habit, hormone replacement therapy use and genetic factors have been implicated in the pathogenesis of cervical cancer[1]. Whereas accumulating evidence suggests that somatic mutations including single nucleotide polymorphisms (SNPs) in tumor suppressor genes and oncogenes play an important role in the genetic susceptibility to cervical cancer[2–4]. Although many publications have focused on the cancer-associated SNPs located in protein-coding genes; several SNPs located in chromosomal regions that do not encode genes may be related to the risk of different cancers.

Long non-coding RNA (lncRNA) is a type of RNAs transcripts that are greater than 200 nucleotides in length and no protein-coding capacity. Although lncRNA play key roles in multiple biologic processes[5–7], several studies reported that they have an important function in carcinogenesis[8–11]. As one of these RNAs, Hox transcript antisense intergenic RNA (HOTAIR) located on chromosome 12q13.13, which is involved in the carcinogenesis of multiple cancers such as breast cancer[12], colon cancer[13], lung cancer[14], esophageal cancer[15] and cervical cancer[16]. They could interact specifically with polycomb repressive complex 2 (PRC2) to target the HOXD locus and apply repressive histone modifications, subsequently reprograming the expression pattern of genes from this locus[17–19]. At this time, according to several studies published to date, SNPs located in the lncRNA HOTAIR locus showed highly significant association with the susceptibility of a variety of human cancers[20–23]. For example, Zhang et al. reported that three haplotype-tagging SNPs (htSNPs) of HOTAIR may influence lncRNA regulation and, thus, is correlated with the risk of esophageal squamous cell carcinoma (ESCC). Additionally, association studies have identified that genetic variants within the HOTAIR gene confer susceptibility to the gastric cancer. However, to our knowledge, no study to date has explored the effect of the polymorphisms in HOTAIR on cervical cancer risk.

In the present study, we hypothesized that genetic variants in HOTAIR could modulate cervical cancer susceptibility. A total of 510 cervical cancer patients and 713 controls were genotyped to assess the associations between three htSNPs (rs920778, rs1899663, and rs4759314) and the risk of the cervical cancer in a Chinese population.

## Subjects and Methods

### Subjects

This study involving 510 cervical cancer patients (cases) and 713 cancer-free individuals (controls) was approved by the Medical Ethics Committee of Second Affiliated Hospital of Soochow University. Patients with cervical cancer were consecutively recruited from the Second Affiliated Hospital of Soochow University, without restriction regarding age as previously described[24]. Each patient was confirmed by a pathological examination at the time of study enrolment. 713 unrelated healthy control subjects matched to the cases with regard to age were randomly recruited from physical examinations from the same geographical region, with a >90% response rate. And these selected controls declared no history family of malignancy. All participants were genetically unrelated Chinese and have given written informed consent. Clinical data was obtained from face-to-face interviews by professional interviewers. In addition, 91 paired cervical cancer tissues and their adjacent normal tissues were obtained from patients with cervical cancer undergoing surgery and were frozen immediately at -80°C until use.

### Cell culture

Two human cervical cancer cell lines SiHa (squamous cervical carcinoma), HeLa (epitheloid cervical carcinoma) were purchased from the Cell Bank Type Culture Collection of the Chinese Academy of Sciences (Shanghai, China). These cells were grown in RPMI-1640 medium supplemented with 10% fetal bovine serum and culture in humidified incubator under 37°C in the presence of 5% CO_2_.

### DNA extraction and genotyping analysis

Genomic DNA was isolated from peripheral blood lymphocytes of all participants. Based on published association studies[20, 23], we selected three htSNPs (rs920778, rs1899663, and rs4759314) polymorphisms in the HOTAIR gene. Briefly, the HapMap public database (HapMap Data Rel 28 PhaseII+III, Auegest10, on NCBI B36 assembly, dbSNP b126) were used to analyze the polymorphisms of HOTAIR gene globally. Moreover, haploview version 4.2 software was selected to determine the HapMap tagSNPs (htSNP) with the criteria of minor allelic frequencies>0.05 in the Chinese populations. Genotyping were performed by using allele specific MALDI-TOF mass spectrometry. To ensure the accuracy of the genotyping, 10% samples without knowing the subjects’ case or control status were randomly subjected to DNA sequencing again, and the results were 100% concordant.

### Plasmid constructs and dual luciferase reporter assay

A portion of 430bp of lncRNA HOTAIR intronic enhancers region either with rs920778C allele or T allele were cloning into pGL3 basic vectors. Transient transfections in SiHa, HeLa and dual luciferase reporter assays were conducted as described earlier [25]. The assays were performed in triplicate, and the results were represented as means ± standard errors of the means under the same conditions.

### RNA isolation and real-time PCR (RT-PCR)

Total RNA from cervical cancer cell lines or frozen human cervical cancer tissues were extracted using TRIzol reagent (Invitrogen) following the manufacturer’s instructions. 1 μg RNA was reverse transcribed into cDNA using the Prime ript RT Master Mix (Takara). HOTAIR and GAPDH expression levels were determined by real-time PCR using an ABI 7500 Real Time PCR System (Applied Biosystem, Foster City, CA). The primers were designed as follows. For HOTAIR, the forward primer was 5’-gctgctccggaatttgagag-3’ and reverse primer was 5’-tgctgccagttagaaaagcg-3’. Relative gene expression level of lncRNA HOTAIR mRNAs was analyzed using the 2-∆∆CT method, normalized to GAPDH mRNA levels.

### Statistical analysis

All statistical analyses were performed using the SAS statistical software package (version 9.3; SAS Institute) and STATA statistical software (version 10.1; StataCorp, College Station, TX, USA). The differences in the distributions of selected demographic variables between cases and controls were evaluated using χ^2^ test, as appropriate. We performed a goodness-of-fit χ^2^ test separately for each SNP to compare the expected genotype frequencies with observed genotype frequencies in controls by the Hardy-Weinberg equilibrium (HWE). Associations between the cervical cancer risk and genotypes were estimated by odds ratios (OR), 95% confidence intervals (CIs) and corresponding *P* values from logistic regression analyses. Paired t test was conducted for group comparison of the HOTAIR expression in cervical cancer tissues and in corresponding normal tissues. One-way analysis of variance was used to evaluate the effect of rs920778C allele or T allele on the luciferase reporter levels and HOTAIR mRNA levels in cervical cancer cell lines and cervical cancer tissues. PS software was used to calculate the statistical power (available at: http://biostat.mc.vanderbilt.edu/twiki/bin/view/Main/PowerSampleSize, January 2015). A *P*<0.05 was used as the criterion of statistical significance.

## Results

### HOTAIR rs920778 polymorphism in cervical cancer

A total of 510 cervical cancer patients and 713 cancer-free individuals were analyzed. Patient data on detailed demographical data and clinical information were collected through questionnaire interview and were presented in Table 1. Over all, there were no significant differences between patients and controls in terms of age, drinking and smoking status. Genotype frequencies of HOTAIR SNPs among the controls were found to be in agreement with the Hardy–Weinberg equilibrium (*P*>0.05 for all). As shown in Table 2, a statistically significant association was found between the HOTAIR rs920778 polymorphism and the risk of cervical cancer (*P*<10^−4^) by logistic regression analysis adjusted for age, smoking status, drinking status and family history of cancer. Patients with the TT+CT genotypes compared with women with the CC genotype in the rs920778 polymorphism had an increased risk of risk of cervical cancer (adjust OR = 1.51, 95%CI = 1.21–1.91, *P* = 0.0004). Also, the T variant allele frequency for the rs920778 polymorphism in patients was 28.73% and was associated with an increased risk of cervical cancer in a dose-dependent manner (*P*<10^−4^). Subsequently, we performed a stratified analyses of rs920778 polymorphism, and the risk effect for rs920778 appeared to be more prominent in the subset of patients with tumor stage (advanced i.e., stage II+III+IV). Compared with the wild-type CC genotype, carriers with advanced stage with variant genotypes of rs920778 (CT+TT) had a 2.17-fold increased risk for developing cervical cancer (adjusted OR = 2.17, 95% CIs = 1.58–2.89, *P* = 0.0005) Table 3. There were no significant differences in genotype frequencies in cases and control for the rs1899663 or rs4759314 polymorphisms in HOTAIR.

**Table 1:** Demographic characteristic of patients with cervical cancer and controls.

	Patients		Controls	
Clinical parameters	N.	(%)	N.	(%)
**Age of diagnosis (mean±SD)**	43±7.3		44±9.1	
**Age (year)**				
≤45	263	(51.6)	344	(48.2)
>45	247	(48.4)	369	(51.8)
**Alcohol consumption**				
Yes	75	(14.7)	94	(13.2)
No	435	(85.3)	619	(86.8)
**Cigarette smoking**				
Yes	31	(6.1)	39	(5.5)
No	479	(93.9)	674	(94.5)
Family history of cancer*				
Yes	37	(7.3)	62	(8.7)
No	473	(92.7)	651	(91.3)
**Histology**				
Adenocarcinoma	25	(4.9)		
Squamous cell	394	(77.3)		
others	91	(17.8)		
**Stage**				
0	71	(13.9)		
I	177	(34.8)		
II	193	(37.9)		
III	67	(13.1)		
IV	2	(0.3)		
**Tumor grade**				
High-moderate	375	(73.6)		
low	78	(15.3)		
unknown	57	(11.1)		
**HPV infection rate**				
Yes	423	(82.9)		
No	87	(17.1)		

*Family history of cancer represents as a history of all human cancers in the parents, brothers and sisters.

**Table 2:** Distribution of allele/genotype frequency of three haplotype-tagging SNPs in HOTAIR and their association with cervical cancer risk.

	Cases		Controls			
Polymorphisms	N = 510	(%)	N = 713	(%)	Adjusted OR^a^ (95%CI)	*P*_value_^b^
**rs920778C>T**						
CC	269	(52.75)	448	(62.83)	1.00 (Reference)	
CT	189	(37.06)	235	(32.96)	1.34 (1.05–1.76)	**<10**^**−4**^
TT	52	(10.20)	30	(4.21)	2.88 (1.76–4.71)	
CT+TT	241	(47.25)	265	(37.17)	1.51 (1.21–1.91)	
Allele						
C	727	(71.27)	1131	(79.31)	1.00 (Reference)	
T	293	(28.73)	295	(20.69)	1.58 (1.31–1.89)	**<10**^**−4**^
**rs1899663G>T**						
GG	356	(69.80)	509	(71.39)	1.00 (Reference)	
GT	146	(28.63)	191	(26.79)	1.07 (0.88–1.45)	0.645
TT	8	(1.57)	13	(1.82)	0.88 (0.35–2.29)	
GT+TT	154	(30.20)	204	(28.61)	1.09 (0.86–1.37)	
Allele						
G	858	(84.12)	1209	(84.78)	1.00 (Reference)	
T	162	(15.88)	217	(15.22)	1.06 (0.84–1.37)	0.654
**rs4759314A>G**						
AA	378	(74.12)	544	(76.30)	1.00 (Reference)	
AG	121	(23.73)	158	(22.16)	1.06 (0.87–1.49)	0.316
GG	11	(2.16)	11	(1.54)	1.41 (0.59–3.58)	
AG+GG	132	(25.88)	169	(23.70)	1.15 (0.91–1.42)	
Allele						
A	877	(85.98)	1246	(87.38)	1.00 (Reference)	
G	143	(14.02)	180	(12.62)	1.17 (0.93–1.41)	0.314

Abbreviations: OR, odds ratio; CI, confidence interval; N, number

^a^Data were calculated by logistic regression analysis adjusted for age, smoking status, drinking status and family history of cancer.

^b^*P*_value_ for Chi-square analysis.

**Table 3:** Stratified analyses of lncRNA HOTAIR polymorphisms with clinical characteristics of patients with cervical cancer.

Variables	Cases (N = 510)				Controls (N = 713)				Adjusted OR (95% CI)^a^	*P*_value_^b^
	CC	(%)	CT/TT	(%)	CC	(%)	CT/TT	(%)	CT/TT *vs*.CC	
**Age(years)**										
≤45	65	(12.75)	198	(38.82)	114	(15.99)	230	(32.26)	1.51 (1.05–2.61)	0.35
>45	204	(40.00)	43	(8.43)	334	(46.84)	35	(4.91)	2.01 (1.25–3.25)	
**Alcohol consumption**										
Yes	43	(8.43)	32	(6.27)	57	(7.99)	37	(5.19)	1.15 (0.62–2.12)	0.34
No	226	(44.31)	209	(40.98)	391	(54.84)	228	(31.98)	1.59 (1.24–2.03)	
**Cigarette smoking**										
Yes	21	(4.12)	10	(1.96)	29	(4.07)	10	(1.40)	1.38 (0.49–3.93)	0.85
No	248	(48.63)	231	(45.29)	419	(58.77)	255	(35.76)	1.52 (1.28–1.91)	
**Family history of cancer**										
Yes	17	(3.33)	20	(3.92)	26	(3.65)	36	(5.05)	0.86 (0.35–1.88)	0.14
No	252	(49.41)	221	(43.33)	422	(59.19)	229	(32.12)	1.66 (1.21–2.04)	
**Histology**										
Adenocarcinoma	11	(2.16)	14	(2.75)	448	(62.83)	265	(37.17)	2.16 (1.01–4.82)	
Squamous cell	210	(41.18)	184	(36.08)	448	(62.83)	265	(37.17)	1.46 (1.13–1.85)	0.68
others	48	(9.41)	43	(8.43)	448	(62.83)	265	(37.17)	1.55 (1.02–2.34)	
**Stage**										
0+I	154	(30.20)	94	(18.43)	448	(62.83)	265	(37.17)	1.02 (0.79–1.36)	
II+III+IV	115	(22.55)	147	(28.82)	448	(62.83)	265	(37.17)	2.17 (1.58–2.89)	**0.0005**
**Tumor grade**										
High-moderate	194	(38.04)	181	(35.49)	448	(62.83)	265	(37.17)	1.57 (1.19–2.07)	
low	43	(8.43)	35	(6.86)	448	(62.83)	265	(37.17)	1.39 (0.88–2.25)	0.78
unknown	32	(6.27)	25	(4.90)	448	(62.83)	265	(37.17)	1.31 (0.73–2.31)	
**HPV infection rate**										
Yes	219	(42.94)	204	(40.00)	448	(62.83)	265	(37.17)	1.55 (1.20–2.00)	
No	50	(9.80)	37	(7.25)	448	(62.83	265	(37.17)	2.24 (1.47–3.57)	0.16

Abbreviations: CI, confidence interval; OR, odds ratio.

^a^The analysis was adjusted by age, smoking, drinking status and family history of cancer. Values in boldface indicate statistical significance.

^b^*P* value for heterogeneity.

### The HOTAIR rs920778 SNP T variant allele located in HOTAIR intron 2 confers enhanced enhancer activity

**Fig 1:**
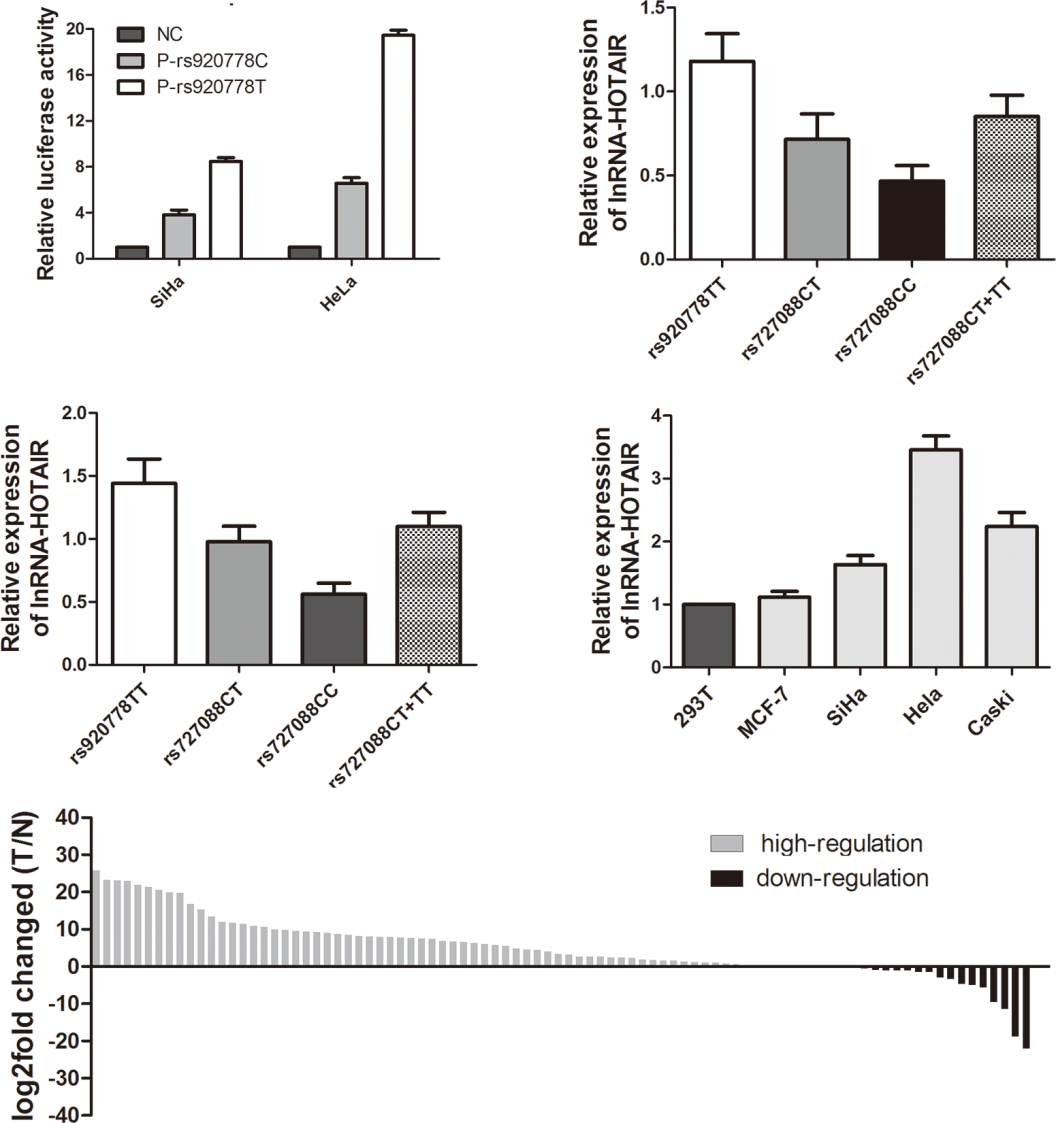
LncRNA-HOTAIR rs920778 SNP T variant allele exhibits enhanced enhancer activity and increases HOTAIR expression. **(A)** Enhancer activity are presented as fold increase relative to negative control (NC, empty vector PGL3). “**” indicates *P*<0.01. Individuals with the risk allele T at rs920778 in both 38 normal cervical tissues **(B)** and 43 cervical cancer tissues **(C)** is significantly associated with increased HOTAIR expression than those with the CC genotypes. Expression levels of HOTAIR are detected by RT-PCR in cervical cancer tissues grouped to three genotypes (rs920778TT, rs920778CT or rs920778CC). “*” indicates *P*<0.05. **(D)** RT-PCR analysis of HOTAIR expression levels in cervical cancer cell lines (SiHa, Hela and Caski) compared with breast cancer cell MCF-7 and human embryo kidney epithelial cell 293T. **(E)** The HOTAIR expression level was analyzed by RT-PCR in 91 cervical cancer tissue samples. HOTAIR expression levels was significantly upregulated in cervical cancer tissues compared with their matched non-tumor cervical cancer tissues (*P*<0.05). T, tumor; N, no-tumor.

Published studies have provided data that there might be a potential enhancer in HOTAIR intron 2 region containing rs920778 SNP. Thus, to test the functional effect of this SNP in enhancer region, the reporter constructs containing either HOTAIR rs920778C or T alleles were transfected into cervical cancer cell lines to determine enhancer activity. We observed substantial increase in luciferase expression for the vectors containing rs920778T allele compared with the C allele in SiHa cells. Similarly to the significantly change expression levels of luciferase observed in SiHa cells, luciferase expression was observed when these constructs were transfected into HeLa cells, with an approximately 3-fold increase for the rs920778T allele than for the rs920778C allele (*P*<0.01) (Fig 1A).

### Association of HOTAIR mRNA expression level and HOTAIR rs920778 polymorphism

A positive correlation of HOTAIR rs920778 polymorphism with the risk of human cancers has been analyzed extensively, but the association between HOTAIR expression and the different genotype of rs920778 polymorphism in cervical cancer was not investigated. As shown in (Fig 1B), real-time PCR assay in the 38 normal cervical tissues revealed that HOTAIR mRNA expression level (mean±standard error) increased with the presence of the risk allele T at rs920778 than those with the CC genotypes [0.715±0.151(n = 12) for CT genotypes and 1.180±0.164 (n = 5) for TT genotypes versus 0.467±0.093 (n = 21) for CC genotypes; *P* = 0.01]. Similar results were also observed in 43 cervical cancer tissues from cervical cancer patients with the HOTAIR polymorphic rs920778 CT+TT genotypes (Fig 1C). Cervical cancer patients with rs920778 CT and TT genotypes exhibited a significantly higher HOTAIR mRNA expression level than individuals having the SNP rs920778CC homozygous genotypes [0.978±0.123 (n = 14) for CT genotypes and 1.441±0.193 (n = 5) for TT genotypes versus 0.560±0.089 (n = 24) for CC genotypes, *P*<0.001].

### HOTAIR expression is increased in cervical cancer cell lines and clinical cervical cancer tissues

**Fig 2:**
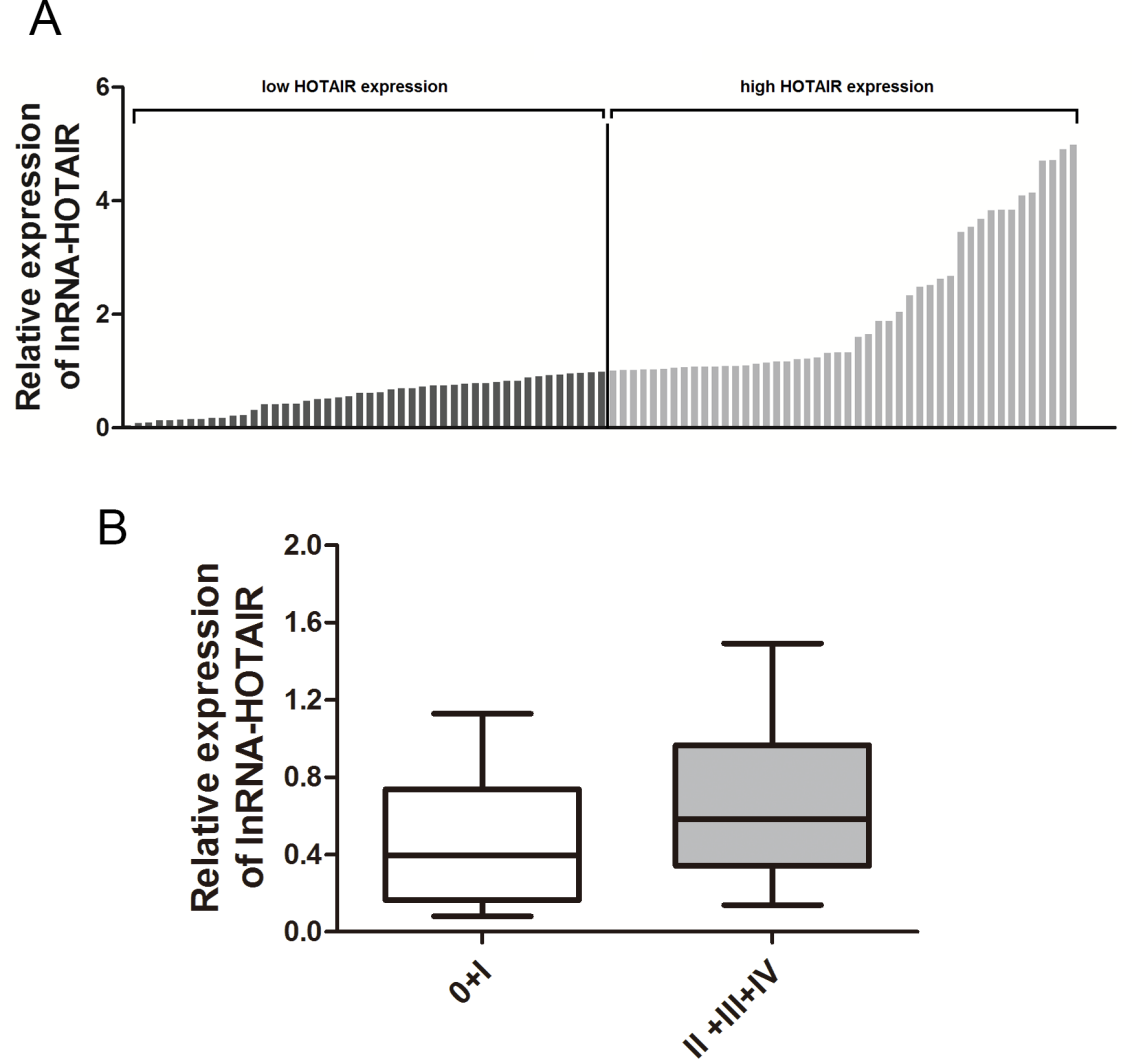
High expression of lncRNA HOTAIR in cervical cancer tissues is associated with patients with tumor node metastasis (TNM) staging. **(A)** HOTAIR expression was classified into two groups based on median value of HOTAIR expression in cervical cancer tissues. **(B)** The HOTAIR expression was significantly higher in patients with advanced pathological stage (II+III+IV) than those with lower pathological stage (0+I).

Considering the interactions between the HOTAIR rs920778 polymorphism genotypes and the increased HOTAIR expression level. We further analyzed HOTAIR expression levels in 3 cervical cancer cell lines and 91 pairs of cervical cancer tissues and the adjacent noncancerous tissues. Confirming previous observations, the expression levels of HOTAIR in cervical cancer cell lines, SiHa, HeLa and Caski cells were significantly higher than breast cancer cells MCF-7 and human embryonic kidney cells 293T as references (Fig 1D). In addition, we sought to evaluate the HOTAIR expression level in cervical cancer tissues and observed that HOTAIR expression level was increased in 72.5%, was unchanged in 8.8%, or was decreased in 18.7% of cervical cancer tissues compared with adjacent tissues (*P*<0.05) (Fig 1E), suggesting that the expression of HOTAIR is upregulated in cervical cancer. According to the median ratio of relative HOTAIR expression (HOTAIR/GAPDH ratio of 0.975) in tumor tissues, we divided the 91 patients with cervical cancer into a high HOTAIR expression group (n = 46) and a low expression group (n = 45) (Fig 2A). We further analyzed the correlation between lncRNA-HOTAIR expression and patient clinicopathological characteristics. As shown in (Fig 2B), high HOTAIR expression in cervical cancer was significant correlation with high TNM stage (II+III+IV) (*P*<0.05). The results indicate that aberrant expression of lncRNA HOTAIR was consistent with the previous report in cervical cancer[26, 27].

## Discussion

Based on previous studies that have identified multiple functional polymorphisms in lncRNA HOTAIR associated with altered expression of HOTAIR and contributing to human cancer risks, we evaluated whether a specific genotype of HOTAIR SNP rs920778 within intronic enhancer plays a pivotal role in cervical cancer. Our study provides extensive experimental evidences to examine the biologic relevance of the HOTAIR SNP rs920778 and found a significant association between the HOTAIR SNP rs920778 polymorphism and cervical cancer susceptibility. Carriers with the variant T genotypes of rs920778 (TT+TC) has been shown to increase approximately 2.88- and 1.34-fold cervical cancer risk than homozygous C allele carriers. In addition, there is strong *in-vitro* experimental evidences linking the rs920778T allele to enhanced expression levels of HOTAIR. Our data, together with the previous data suggest that the particular HOTAIR SNP rs920778 contributes to the cervical cancer risk.

It is becoming evident that a broad spectrum of human lncRNAs transcribed by mammalian genomes have been extensively studied and their roles in diverse cellular processes, physiology and diseases [28–30]. In the last years, evidence has begun to accumulate highlighting the molecular mechanisms by which these transcripts exerts their function. One well-studied mechanism is RNA-mediated transcriptional silencing by preventing recruitment of the initiation complex or transcriptional elongation[31]. Other lncRNAs may act as co-activators to activate nearby genes transcriptional activity by binding to transcription factors[32–34]. In addition, several lncRNAs have also been shown to interact with chromatin-modifying complexes, to regulate target genes expression [35, 36]. Based on their roles, the dysregulation of lncRNAs have been proved to be involved in development and progression of various types of cancers [37–39]. As one of the well-studied lncRNAs, studies have shown that disruption of lncRNA HOTAIR action occurs in different cancer types, such as lung cancer[40], breast cancer [41] and nasopharyngeal carcinoma [42]. The association between HOTAIR and cervical cancer has also demonstrated that upregulation of HOTAIR promoted cervical carcinoma cells proliferation, invasion and migration [27, 43]. In addition, in a meta-analysis containing eight eligible studies, zhang et al. [44] showed that HOTAIR may be an indicator of poor prognosis in four main estrogen-dependent tumors, including cervical, ovarian, breast and endometrial cancers. The study of HOTAIR expression in the sera of cervical cancer patients indicate that the level of circulating HOTAIR may serve as a promising predicting and therapeutic target in cervical cancer[26]. To date, based on the HapMap database, SNPs in the HOTAIR gene (http://www.ncbi.nlm.nih.gov/snp/) have been reported to affect its expression, and consequently influence risks of human cancers[20–23]. One such SNP is rs920778, whose associations with cancer susceptibility have been reported by a number of studies. In this study, we found a significant relation between HOTAIR rs920778 genotypes and cervical cancer risk. Moreover, the TT genotype of rs920778 was significantly associated with advanced TNM (II+III+IV) classification. Consisted with previously published study on the biology of HOTAIR in cervical cancer, in our study, it is generally showed that HOTAIR played a critical role in cervical cancer. HOTAIR expression was higher in cervical cancer tissues than that in corresponding normal tissues, and the high expression was associated with the TT genotype of rs920778. These lines of evidence suggest that the TT genotype of SNP rs920778 in the HOTAIR gene may play crucial roles in the development of cervical cancer by influencing HOTAIR expression. However, our data in the current study have some potential limitations. Selection bias may occur, because it was a hospital-based case–control study that the cases were from hospitals and the controls were from the community. Furthermore, relatively larger sample size should be required to further validate the associations of these SNPs with cervical cancer risk.

Taken together, our study suggested that SNPs in the lncRNA HOTAIR might contribute to the susceptibility of cervical cancer. Further large-scale studies in different populations are needed to verify our results.
